# Highly Dispersed CoO Embedded on Graphitized Ordered Mesoporous Carbon as an Effective Catalyst for Selective Fischer–Tropsch Synthesis of C_5+_ Hydrocarbons

**DOI:** 10.3389/fchem.2022.849505

**Published:** 2022-02-10

**Authors:** Jirong Bai, Mingyao Song, Jiazheng Pang, Lingling Wang, Jianping Zhang, Xiankai Jiang, Zhijiang Ni, Zhilei Wang, Quanfa Zhou

**Affiliations:** ^1^ Research Center of Secondary Resources and Environment, School of Chemical Engineering and Materials, Changzhou Institute of Technology, Changzhou, China; ^2^ Department of Wood Science, The University of British Columbia, Vancouver, BC, Canada; ^3^ School of Mechanical Engineering and Urban Rail Transit, Changzhou University, Changzhou, China; ^4^ Department of Environmental Science and Engineering, Fudan University, Shanghai, China

**Keywords:** Fischer–Tropsch synthesis, clean soybean oil, cobalt catalyst, order mesoporous carbon, active carbon

## Abstract

Herein, we report the high Fischer–Tropsch synthesis performance of the Co-based catalysts supported on graphitized ordered mesoporous carbon (GMC-900) by using a facile strategy. Compared with CMK-3 and active carbon (AC), the obtained GMC-900 by using pollution-free soybean oil as a carbon source exhibited enhanced catalytic performance after loading Co species due to its highly crystallized graphitic structure and uniform dispersion of CoO. As a result, Co/GMC-900 was an effective catalyst with the maximum C_5+_ selectivity of 52.6%, which much outperformed Co/CMK-3 and Co/AC. This research provides an approach to produce advanced Co-based catalysts with satisfactory performance for efficient Fischer–Tropsch synthesis.

## Introduction

With the rapid development of human society and economy, it becomes more and more difficult for crude oils to meet people’s demands (Guo et al., 2016; Hao et al., 2016; Zhang et al., 2017). Furthermore, the serious problems of air pollution and greenhouse effect caused by the consumption of fossil fuels have impelled the search for more clean alternatives (Qin et al., 2016). Fischer–Tropsch synthesis (FTS) is an alternative route that can transform synthesis gases resulting from gasification of biomass, natural gases, and coals into clean liquid fuels or chemicals without containing nitrogen, sulfur, or aromatics (Liu et al., 2018; Lyu et al., 2018). Traditionally, Ru-based catalysts exhibit high low-temperature selectivity and activity for long-chain hydrocarbons (Guo et al., 2019). However, the large-scale commercial application in industry is severely hampered by some major problems, especially the scarcity in nature and expensive prices (Liu et al., 2018; Guo et al., 2019). In comparison, Co-based catalysts, one of the most optimal choices for FTS due to their low costs, are highly active and selective toward long-chain hydrocarbons, and possess water deactivation stability and low water–gas shift activity during the reaction (Johnson and Bell, 2016; Cheng et al., 2018).

The support of Co-based catalysts greatly influences the catalytic performance during the reaction (Fu and Li, 2015; Qin et al., 2016). Conventionally, various oxide materials such as SiO_2_, Al_2_O_3_, and TiO_2_ are used to support Co-based catalysts. SiO_2_ and Al_2_O_3_ are preferentially used owing to their large surface areas, abrasion resistance, and excellent mechanical properties (Bustamante et al., 2020; Wolf et al., 2020; Xu et al., 2020). However, the relatively strong interaction between cobalt oxide and the support leads to the generation of irreducible substances, such as CoAl_2_O_4_ or Co_2_SiO_4_ (Bustamante et al., 2020; Li et al., 2020). As a result, the number of surface active sites on the support decreases, which limits the activity for long-chain hydrocarbons (Wang et al., 2020). Therefore, exploring suitable Co-based catalysts with the low formation of hardly reducible materials is highly desirable.

Carbon materials are also considered as some of the most desirable supports in FTS owing to their unique specific structure and chemistry, such as surface inertness, controllable crystallized graphitic structure, high conductivity, and acid or alkali resistance (Lyu et al., 2019; Dlamini et al., 2020). Reuel and Bartholomew (1984) found that the catalytic performance in CO hydrogenations dropped considerably with the increase of cobalt dispersion degree as follows: Co/TiO_2_ > Co/SiO_2_ > Co/Al_2_O_3_ > Co/C > Co/MgO. Tavasoli et al. (2008) reported that Co/CNT catalysts prepared from the impregnation method exhibited much larger activity than Co/Al_2_O_3_ on hydrocarbon yield, which can be ascribed to the weak interaction between Co species and CNT. In addition, mesoporous carbon (MC) using mesoporous silica as a precursor has also received much attention on FTS owing to its ordered pore structure and large surface area (Wu et al., 2019). Zhao et al. (2020) prepared Co/MC-1300 (where MC was prepared from pyrolysis of furfuryl alcohol and SBA-16 as the hard template), which showed improved cobalt reducibility and C_5+_ selectivity up to 74%.

The structure of carbon supports, particularly the porous structure, greatly influences the FTS performance (Xiong et al., 2011). Nonetheless, the impact of different carbon sources as the support on the structure and catalysis in FTS is rarely reported. Herein, we report the high Fisher-Tropsch synthesis performance of the Co catalysts supported on graphitized ordered mesoporous carbon (GMC-900) by using pollution-free soybean oil as a carbon source. We also chose the porous CMK-3 prepared and commercial AC from other carbon sources as the supports and controls to evaluate the catalytic performance of GMC-900 in FTS.

## Experimental

### Chemicals

Poly-(ethylene glycol)-block-poly-(propylene glycol)-poly-(ethylene glycol) (P123, average Mn ∼5,800) was supplied from Sigma-Aldrich. Ethyl silicate (TEOS, SiO_2_ ≥ 28%) was purchased from Shanghai Lingfeng Chemical Reagent Co., Ltd., China. Active carbon (AC) was obtained from Huajing Activated Carbon Co., Ltd., China. CMK-3 was supplied from Nanjing Xianfeng Nanomaterials Technology Co. Ltd., and soybean oil was obtained from Jiangsu Junqi Grain and Oil Co., Ltd. Ethanol, glycerol, hydrochloric acid, sodium hydroxide, cobalt nitrate, and dicyandiamide were obtained from Sinopharm Chemical Reagent Co., Ltd. (China). Nitrogen (99.5%), argon (99.999%), hydrogen argon mixed gas (V_H2_/V_Ar_ = 5/95), and syngas (V_H2_/V_CO_/V_Ar_ = 64:32:4) were purchased from Shanghai Pujiang Gas Co., Ltd. (China). All chemicals were used as received.

### Sample Preparation

#### Synthesis of Mesoporous Molecular Sieve SBA-15

Typically, 6 g of the mixture of P123 and glycerol with the same mass ratio was dispersed into 115 g of hydrochloric acid aqueous solution (M_HCl_ = 1.5 M) under vigorous stirring at 37°C for 3 h. Then, 6.45 g of ethyl silicate (TEOS) was added dropwise under vigorous stirring. After 5 min, the resulting mixture was kept static for 24 h, and then the obtained mixture was treated with a drying box at 110°C for 12 h. The dried product was gathered by filtering and washing, and dried at 80°C overnight. Finally, SBA-15 was obtained *via* calcination at 550°C for 5 h to get rid of the surfactant.

#### Synthesis of GMC-900

GMC-900 was prepared by a simple solid–liquid grinding/templating route and calcination using SBA-15 as a hard template and soybean oil as a carbon source. In a typical synthesis, the mixture of SBA-15 and soybean oil with a mass ratio of 1:2 was ground together homogeneously under ball milling (400 rpm min^−1^) for 5 h; then the mixture obtained was transferred into a quartz boat and calcined at 900°C for 5 h (heating ramp 4°C min^−1^) in nitrogen gas. Finally, the obtained product was treated by NaOH aqueous solution etching to remove SBA-15, and then filtered and dried to collect GMC-900.

#### Synthesis of Co/GMC-900, Co/CMK-3, and Co/AC

In general, Co/C was prepared using the impregnation method. Briefly, 0.8 g of GMC-900 (or CMK-3 or AC) was added into a cobalt nitrate ethanol solution (0.8719 g, Co (NO_3_)_2_·6H_2_O) under stirring, and stirring was continued for 1 h. Then the cobalt nitrate ethanol solution was evaporated at 35°C in a rotary evaporator. Finally, the obtained mixture was dried at 50°C for 12 h, and then carbonized at 350°C in nitrogen gas. The products were marked as Co/GMC-900, Co/CMK-3, and Co/AC, respectively.

### Characterizations

The microstructure of materials was characterized by a JEOL JEM-2010 transmission electron microscope (TEM, 200 kV). The Barrett–Joyner–Halenda (BJH) pore size distributions and Brunauer–Emmett–Teller (BET) specific surface areas were measured by N_2_ ad-/desorption isotherms. X-ray diffraction (XRD) was measured on a Rigaku D/Max2rB-II device (Cu Kα radiation, *λ* = 1.5406 Å) at a rate of 4°min^−1^ from 20 to 90°. Thermogravimetric analysis (TGA) was carried out by a TGA 8000 analyzer by heating to 900 °C at a rate of 10°C min^−1^. Raman spectra were observed on a Dilor Labram-1B spectrometer with a 632-nm laser. The behaviors of the samples in H_2_ temperature-programmed reduction (TPR) were investigated on a home-made instrument with a thermal conductivity detector (TCD).

### Catalysis Measurement

Catalytic performances were assessed using a tubular fix-bed reactor at 270°C, *p* = 2 MPa, and H_2_/CO = 2. Briefly, the isothermal zone of the reactor was placed with a catalyst (0.3 g) blended with quartz granules (40–60 meshes, 2.4 g), and its remaining part was filled with the quartz granules. The reactor was maintained at 450°C and 0.4 MPa in H_2_ atmosphere (H_2_/Ar = 5/95, v/v) for 16 h. After *in situ* catalyst reduction, the reactor was cooled to 120°C in H_2_ atmosphere. Then the syngas H_2_/CO/Ar (64:32:4) flowed at a rate of 30 ml min^−1^ (GHSV = 3.6 L h^−1^ g^−1^) through the catalysis bed at 2 MPa and 270°C. Unreacted gases (H_2_, N_2_, and CO) and by-products (CH_4_ and CO_2_), and hydrocarbons (C_1_–C_30_) can be detected by TCD and flame ionization detector, respectively.

## Results and Discussion

**FIGURE 1:**
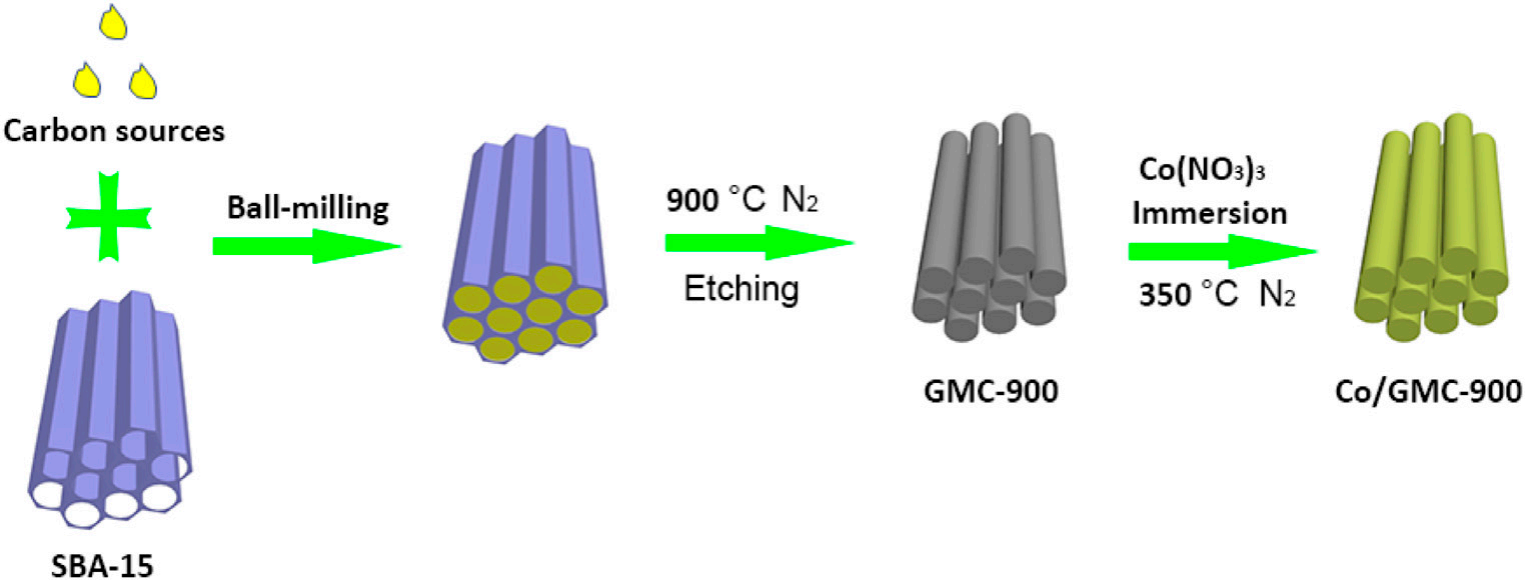
Schematic scheme for synthesis of Co/GMC-900.

The procedure for Co/GMC-900 preparation is demonstrated in Figure 1. In brief, SBA-15 was synthesized in an acidic environment with ethyl silicate as the silicate source and P123 as the soft template. Subsequently, the obtained SBA-15 was mixed with edible soybean oil through ball-milling to form a homogeneous mixture. Then GMC-900 was collected through carbonizing and etching the above mixture. Finally, Co (NO_3_)_3_ and GMC-900 were mixed by immersion and heat treatment to form Co/GMC-900.

**FIGURE 2:**
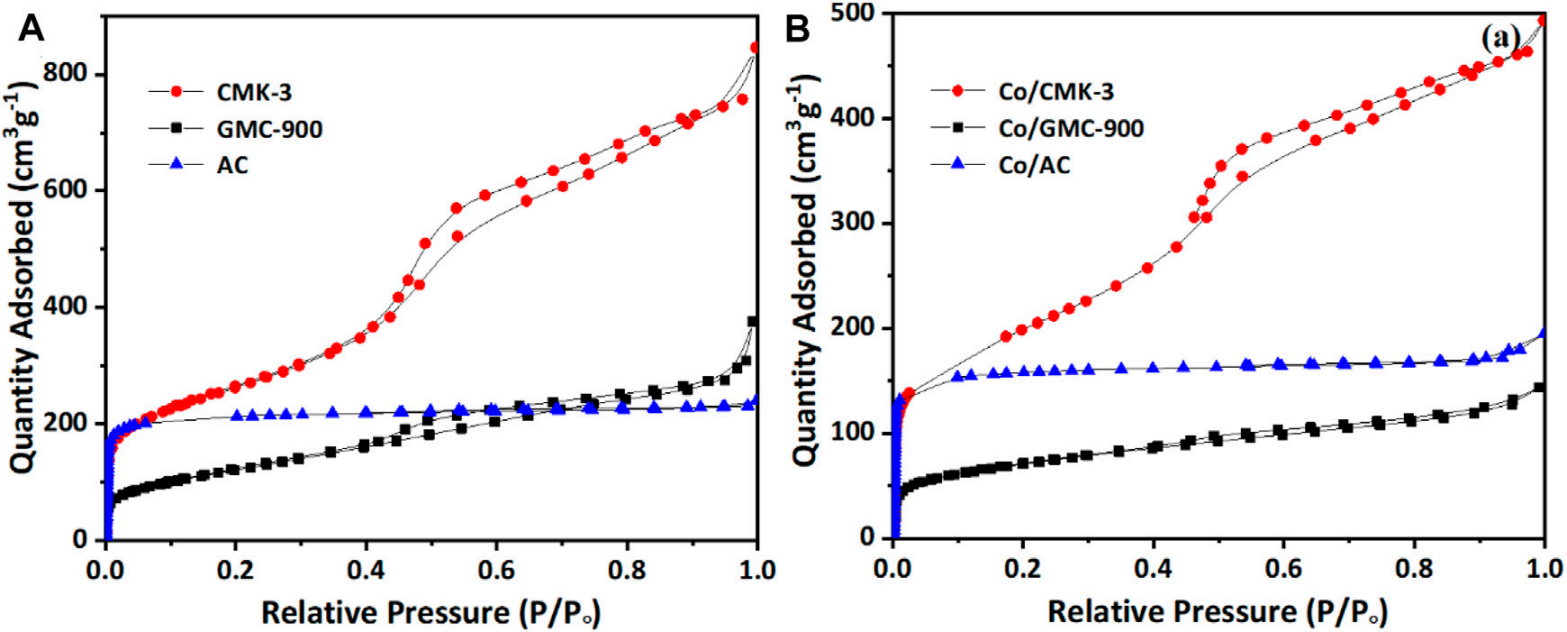
The N_2_ adsorption–desorption isotherms of CMK-3, GMC-900, and AC **(A)**, and Co/CMK-3, Co/GMC-900, and Co/AC **(B)**.

N_2_ ad-/desorption isotherms used to explore the specific area before and after cobalt loading are displayed in Figure 2. Both GMC-900 and CMK-3 correspond to class IV curves with a typical hysteresis loop at relative pressure P/P_0_ of 0.4–1.0 (Figure 2A), which suggests the existence of a mesoporous structure (Zhao et al., 2020). The ad-/desorption isotherm of AC is a class I curve, which is characteristic of microporous structures (Asami et al., 2013). After cobalt impregnation, similar ad-/desorption isotherms were found for these catalysts (Figure 2B), suggesting the carbon supports are almost structurally constant (Fu and Li, 2015). The physicochemical properties of carbon supports and catalysts are listed in Table 1. The specific surface areas of CMK-3 and AC (939 and 657 m^2^ g^−1^, respectively) greatly surpass that of GMC-900. However, the average pore diameter of AC (2.25 nm) is smaller than those of CMK-3 and GMC-900. Furthermore, the pore volumes and specific surface areas of catalysts decrease after loading Co species on the carbon supports. The reason for these results is that the adsorption of cobalt oxide particles onto mesoporous walls leads to the blockage of small pores (Khodakov et al., 2002). Compared with other catalysts, the pore volume of AC decreases slightly, resulting from the entrance of micropores being more easily blocked than that of mesopores.

**TABLE 1:** Physicochemical properties of carbon supports and catalysts.

Sample	Surface area (m^2^ g^−1^)	Pore size (nm)	Pore volume (cm^3^ g^−1^)
GMC-900	442	5.24	0.58
CMK-3	939	5.57	1.31
AC	657	2.25	0.37
Co/GMC-900	306	3.51	0.26
Co/CMK-3	709	4.30	0.76
Co/AC	492	2.44	0.30

**FIGURE 3:**
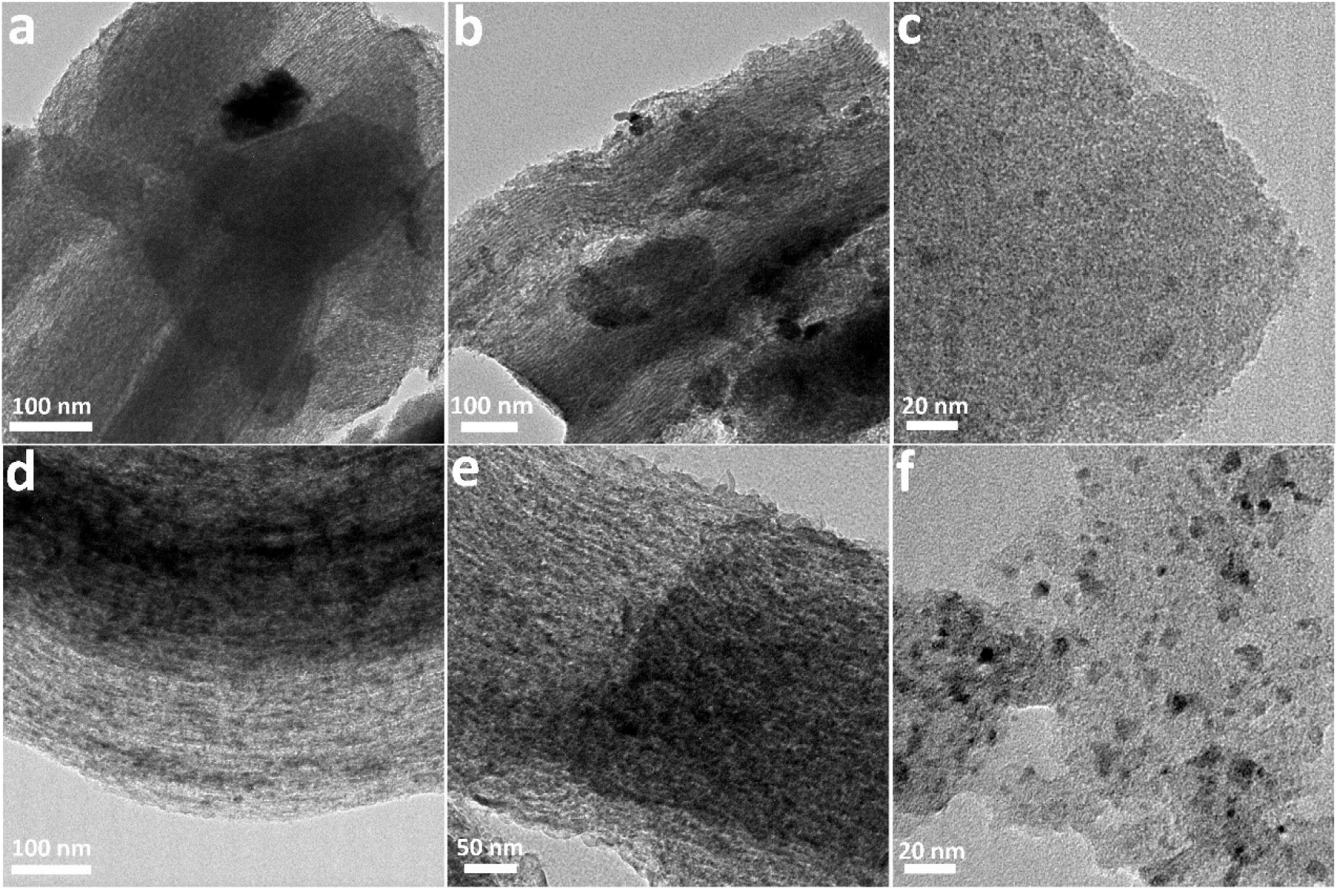
TEM images of CMK-3 **(A)**, GMC-900 **(B)**, AC **(C)**, and the corresponding Co-based catalysts **(D–F)**.

As shown in Figure 3, the TEM images further reveal the typical mesoporous and morphological structures of mesoporous carbon materials and the corresponding Co-based catalysts. Figure 3A exhibits the ordered mesoporous structure of CMK-3. GMC-900 is similar to CMK-3, while the mesoporous structure was damaged slightly due to the high-temperature treatment (Figure 3B) (Hanzawa et al., 2002). After Co species loading, the morphological structures of catalysts are very different. As for Co/CMK-3 and Co/GMC-900, CoO was uniformly dispersed in the mesopores (Figures 3D,E). However, the ordered meso-structure of Co/GMC-900 was partially damaged, which can be due to both high temperature and Co species loading (Fu and Li, 2015). As for Co/AC, some particles appeared due to the aggregation of CoO after Co species loading (Figure 3F).

**FIGURE 4:**
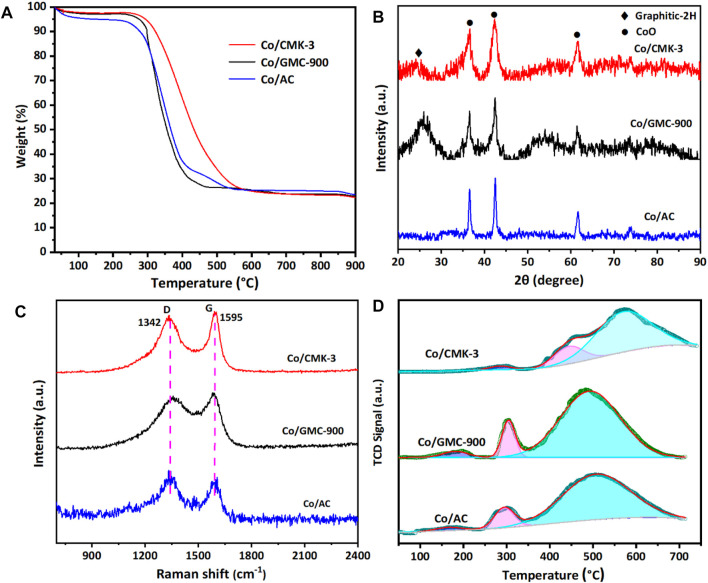
TGA **(A)**, XRD patterns **(B)**, Raman spectra **(C)**, and H2–TPR **(D)** of Co/CMK-3, Co/GMC-900, and Co/AC.

The thermal stability and amount of CoO in the catalysts were analyzed by TGA in air atmosphere (Figure 4A). All the catalysts experienced mass losses of about 76.3% during calcination. The first mass loss below 200 °C was due to the desorption of physically adsorbed water (Liu et al., 2018). The second remarkable mass loss occurs at 240–600 °C, corresponding to the combustion of graphitic carbon (Qin et al., 2016; Sun et al., 2012). The structural characteristics of Co/CMK-3, Co/GMC-900, and Co/AC were determined by XRD patterns (Figure 4B). The peaks at around 36.4, 42.3, 61.6, 73.9, and 77.8° are indexed to the (111), (200), (220), (311), and (222) reflection for the CoO phase (JCPDS no. 43-1004) (Loewert et al., 2020; Xu et al., 2020). The broad peak at around 26.6° can be attributed to the graphitic carbon (JCPDS no. 41-1487) (Qiu et al., 2014). Compared with Co/CMK-3 and Co/AC, the intensity of this strong graphitic peak for GMC-900 indicates a high degree of graphitization (Torshizi et al., 2020). In addition, the strong peaks of CoO in Co/AC suggest that the particle size of CoO is larger than those of Co/CMK-3 and Co/GMC-900, which is in accordance with the TEM image (Figure 3F). Calculation shows that the average particle sizes of CoO in Co/CMK-3, Co/GMC-900, and Co/AC are 6.9, 11.6, and 19.2 nm, respectively.

To further check the carbon structures of the obtained materials, we conducted Raman spectra to recognize the changes of carbon species and the defects in the graphite layer. All catalysts display a D band at around 1342 cm^−1^ and a G band at around 1595 cm^−1^ (Figure 4C). The D band corresponds to a defect in the lattice of carbon atoms, while the G band reflects the change of sp^2^ hybridized carbon atoms in the graphite layer (Trépanier et al., 2009; Vosoughi et al., 2016; Zeng et al., 2021). Generally, the ratio of relative integrated intensity (I_D_/I_G_) between these two bands implies the degree of graphitization and the disorder degree of functionalized groups and defects, which was calculated to be 1.98, 1.55, and 2.65 for Co/CMK-3, Co/GMC-900, and Co/AC, respectively. These results indicate that GMC-900 has a high degree of graphitization than Co/CMK-3 and Co/AC, which are in accordance with the above XRD results.

The reducibility of catalysts can be evaluated by H_2_-TPR measurements (Figure 4D). Obviously, all the catalysts display three partially overlapped peaks. The first and second peaks reflect the reduction of Co_3_O_4_ to CoO, and the reduction of CoO to Co, respectively, and the third reduction peak in the TPR spectra implies the gasification of carbon support (Sun et al., 2012). The third reduction peak around 574°C for Co/CMK-3 is higher than that for Co/GMC-900 and Co/AC, which indicates that it is more irreducible. The absence of the reduction peak above 600°C suggests no formation of hardly reducible substances on the catalyst surface because of the much lower interaction of carbon support with Co species than with traditional oxides (TiO_2_, Al_2_O_3_, or SiO_2_) (Bai et al., 2012; Cheng et al., 2018).

**FIGURE 5:**
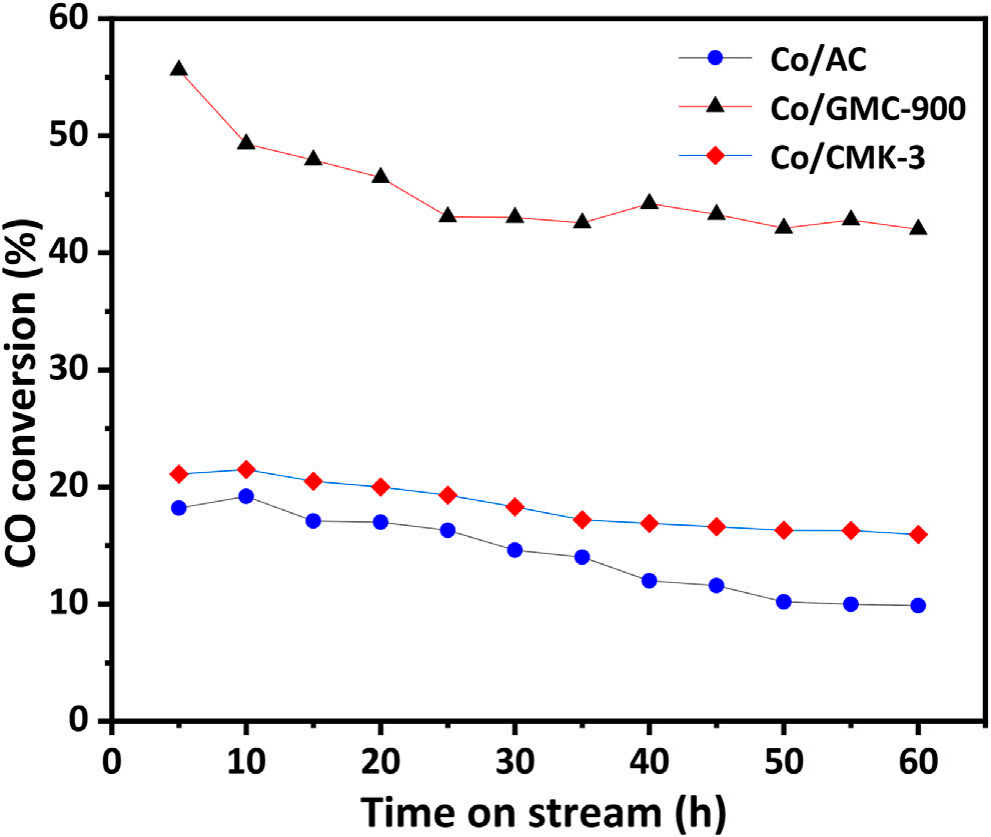
Temporal CO conversion of the catalysts on stream.

Catalytic behaviors of all the catalysts were explored by FTS performed at *p* = 2 MPa, T = 270°C, H_2_/CO = 2, and GHSV = 3.6 L h^−1^ g^−1^. Before reaction, the catalysts were reduced *in situ* in H_2_ flow at 450°C for 16 h. Figure 5 exhibits the temporal CO conversion on stream within a time period of 60 h. Among the catalysts, the Co/GMC-900 exhibits relatively higher CO conversion, and even after 60 h, it still has the CO conversion above 40%. As for the Co/CMK-3, the CO conversion is relatively lower than that of Co/GMC-900, while the catalytic activity is more stable (CO conversion decreasing from 21 to 15%). These results can be explained by its ordered pore structure, large specific surface area and pore volume, and open pore structure, benefiting the adsorption and diffusion of syngas in the catalysts (Ahn et al., 2016). By contrast, the Co/AC exhibits lower CO conversion, which can be ascribed to the poor dispersion of CoO (Figure 3C).

Table 2 lists the results of catalysts in FTS, including the catalytic behaviors and production distribution. These data were the mean values over 60 h. Clearly, Co/GMC-900 has a maximum CO conversion rate of 45.2%, resulting from the more reducible of Co/GMC-900 than that of Co/CMK-3 and Co/AC so that it can offer more active sites for FTS. In addition, the highly crystallized graphitic structure of GMC-900 can accelerate the electron transport between CoO and CO, thus facilitating the activation of CO (Reuel and Bartholomew, 1984; Fan et al., 2009). In comparison, despite the high dispersion of CoO on CMK-3, large specific surface area, and the orderly mesoporous structure, Co/CMK-3 only achieved the CO conversion of 18.1%, which can be attributed to the less crystallized graphitic structure of CMK-3. In addition, compared with Co/CMK-3 and Co/AC, Co/GMC-900 was less selective toward CH_4_ (22.4%) and C_2_–C_4_ hydrocarbons (25.1%) (Table 2), resulting in the increased C_5+_ selectivity of Co/GMC-900. For Co/CMK-3, the higher CH_4_ selectivity can be assigned to smaller CoO particles.

**TABLE 2:** FTS catalytic performance of Co/GMC-900, Co/CMK-3, and Co/AC catalysts.

Catalyst	CO conv. (%)	Selectivity (%)			CO_2_ selectivity (%)
		CH_4_	C_2_–C_4_	C_5+_	
Co/GMC-900	45.2	22.4	25.1	52.6	36.4
Co/CMK-3	18.1	32.1	29.2	24.3	30.7
Co/AC	13.3	43.7	36.0	20.8	88.7

**FIGURE 6:**
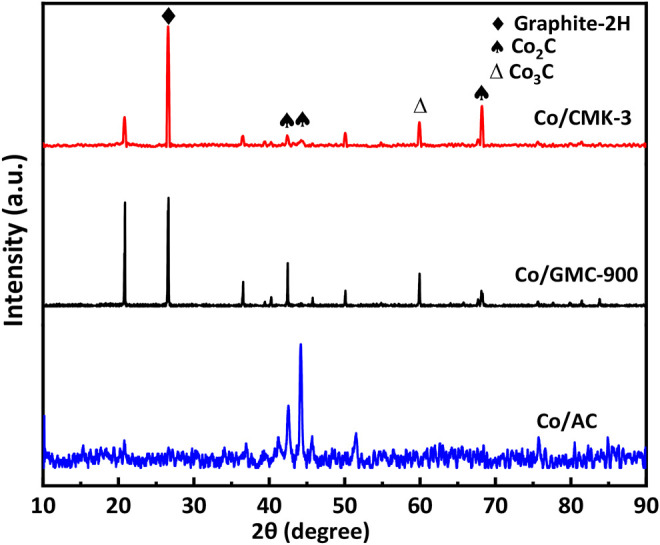
XRD patterns of Co/CMK-3, Co/GMC-900, and Co/AC after FTS for 60 h.

**FIGURE 7:**
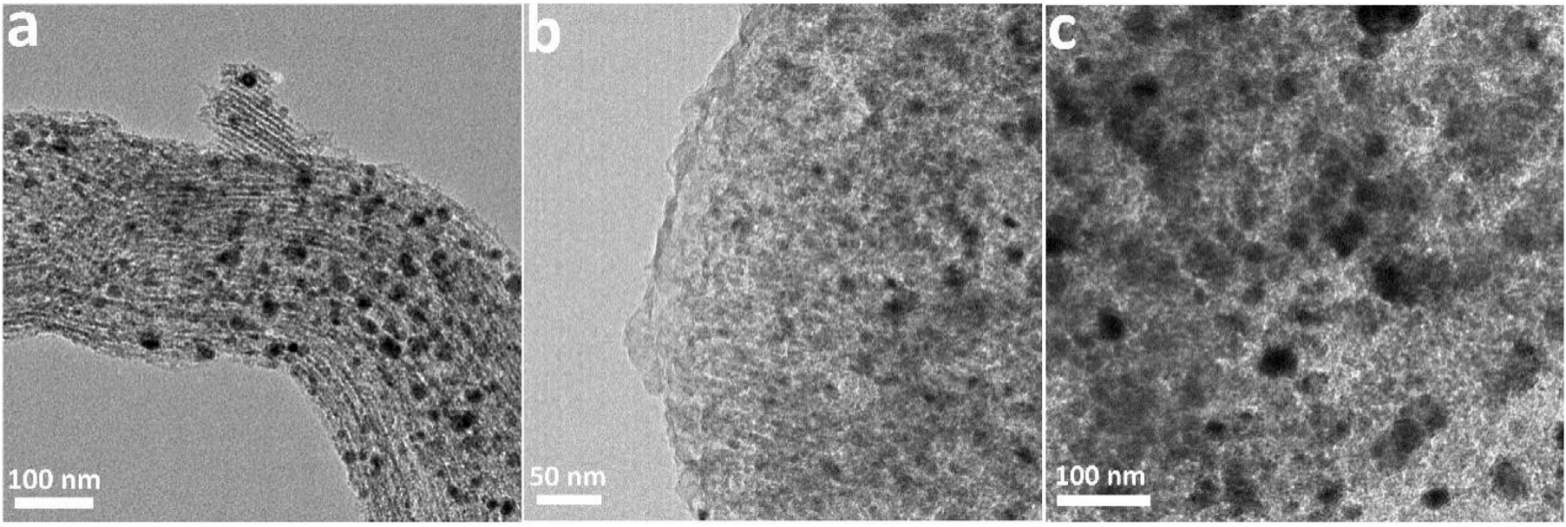
TEM images of Co/CMK-3 **(A)**, Co/GMC-900 **(B)**, and Co/AC **(C)** after 60 h for FTS.

Figure 6 exhibits the XRD patterns of all the catalysts after 60 h for FTS. Three strong diffraction peaks at 42.5, 45.7, and 68.3° in the XRD patterns of these catalysts are assigned to Co_2_C (JCPDS 72-1369) (LÜ et al., 2012). The other diffraction peaks at 60 and 75° correspond to Co_3_C (JCPDS 89-2866) (Liu et al., 2019). These results suggest the appearance of new species of catalysts during FTS, resulting from the decreased conversion of CO. Compared with unreacted catalysts, the peak at around 26.1° in the XRD patterns of used Co/CMK-3 and Co/GMC-900 is stronger, indicating the slight carbon deposition on the catalyst surface (Qin et al., 2019). The aggregation of Co species in catalysts with different sizes was shown in the TEM images (Figure 7). In comparison, Co/GMC-900 has a slight aggregation, resulting in the CO conversion which is decreased slowly than that of Co/CMK-3 and Co/AC. This result is in accordance with the results of Table 2.

## Conclusion

In summary, we demonstrate a simple method to prepare Co-based catalysts supported on graphitized ordered mesoporous carbon (GMC-900) for FTS. Compared with other mesoporous carbon (CMK-3 and AC), the obtained GMC-900 by using pollution-free soybean oil as a carbon source exhibited enhanced catalytic performance after loading Co species due to its highly crystallized graphitic structure and uniform dispersion of CoO. FTS results indicate Co/GMC-900 has high catalytic effectiveness with the largest C_5+_ selectivity up to 52.6%, which greatly surpasses those of Co/CMK-3 and Co/AC. Therefore, our work provides important information to produce high-performance FTS catalysts through ball-milling of clean soybean oil as a carbon source.

## Data Availability

The original contributions presented in the study are included in the article/Supplementary Material; further inquiries can be directed to the corresponding authors.
